# Sleep Duration, Lipid Profile and Insulin Resistance: Potential Role of Lipoprotein(a)

**DOI:** 10.3390/ijms21134680

**Published:** 2020-06-30

**Authors:** Lyudmila Korostovtseva, Asiiat Alieva, Oxana Rotar, Mikhail Bochkarev, Maria Boyarinova, Yurii Sviryaev, Aleksandra Konradi, Eugene Shlyakhto

**Affiliations:** 1 Almazov National Medical Research Centre, St Petersburg 197341, Russia; alieva_as@almazovcentre.ru (A.A.); rotar_op@almazovcentre.ru (O.R.); bochkarev_mv@almazovcentre.ru (M.B.); boyarinova_ma@almazovcentre.ru (M.B.); sviryaev_yuv@almazovcentre.ru (Y.S.); konradi_ao@almazovcentre.ru (A.K.); shlyakhto@almazovcentre.ru (E.S.); 2 Sechenov Institute of Evolutionary Physiology and Biochemistry of RAS, St Petersburg 194223, Russia; 3 Institute of Translational Medicine, ITMO University, St Petersburg 197101, Russia

**Keywords:** sleep, sleep duration, cardiovascular risk, dyslipidemia, lipoprotein (a), insulin resistance

## Abstract

Lipoprotein (a) (Lp(a)) is considered a genetic factor for cardiovascular disease playing an important role in atherogenesis and thrombosis, but the evidence about its association with sleep duration is controversial. We evaluated the relation between self-reported sleep duration and Lp(a). Among 1600 participants of the population-based sample, we selected 1427 subjects without previously known cardiovascular events, who answered the questions about their sleep duration; had valid lipid profile results (total cholesterol, low- and high-density lipoproteins, Lp(a), apolipoprotein AI (ApoAI), ApoB, and ApoB/ApoAI); and did not take lipid-lowering drugs (mean age 46 ± 12 years). We performed a structured interview, which included questions about lifestyle, medical history, complaints, and sleep duration (How long have you been sleeping per night during the last month?). Sleep duration was classified as follows: <6 h/night—short, 6–9 h/night—normal, and ≥10 h/night—long. Overall, 73 respondents (5.2%) were short-sleepers and 69 (4.8%) long-sleepers. Males were slightly more prevalent among short-sleepers. The groups matched by age, body mass index, blood pressure, diabetes mellitus, and hypertension rate. Short-sleepers had lower rates of high total cholesterol (≥5.0 mmol/L), lower Lp(a) levels and lower rates of increased Lp(a) ≥0.5 g/L, and higher insulin and insulin resistance (assessed by the homeostatic model assessment for insulin resistance (HOMA-IR)). ApoAI, ApoB, their ratio, and other lab tests were similar in the groups. The multinomial logistic regression demonstrated that only the short sleep duration was independently (odds ratio (OR) 0.29, 95% confidence interval (CI) (0.09–0.91), *p* = 0.033) associated with Lp(a) (χ^2^ = 41.58, *p* = 0.003). Other influencing factors were smoking and HOMA-IR. Such an association was not found for long-sleepers. In conclusion, a short-sleep duration is associated with Lp(a). The latter might mediate the higher insulin resistance and higher cardiometabolic risks in short-sleepers.

## 1. Introduction

The traditional, well-known cardiovascular risk factors include blood pressure (BP), total cholesterol and low-density lipoproteins, smoking, and sex [1,2]. At the same time, current evidence and guidelines establish that risk stratification can be improved when additional factors are assessed, especially for those at low or moderate risk, according to the traditional risk stratification. A number of factors are suggested as potential modifiers. Among biochemical additional factors, the apolipoprotein B and lipoprotein(a) (Lp(a)) are considered potentially modifiable, albeit less investigated, risk factors for cardiovascular diseases [1,2].

Among behavioral risk factors, sleep and sleep patterns are investigated as important determinants of cardiometabolic health. Sleep quality, sleep duration, and sleep disorders are discussed as potential targets for medical intervention [3]. Epidemiological studies demonstrate that both short and long sleep are associated with unfavorable cardiovascular outcomes (coronary artery disease, stroke, and cardiovascular death) [4,5] and metabolic disorders [6]. However, the evidence about an association between sleep duration and dyslipidemia is rather scarce and contradictory and lacking information about Lp(a) and apolipoproteins [7,8,9,10,11].

The aim of the current analysis was the estimation of the association between self-reported sleep durations and the lipid profile, including Lp(a) and apolipoproteins AI (apoAI) and B (ApoB), in the general population.

## 2. Results

**Figure 1:**
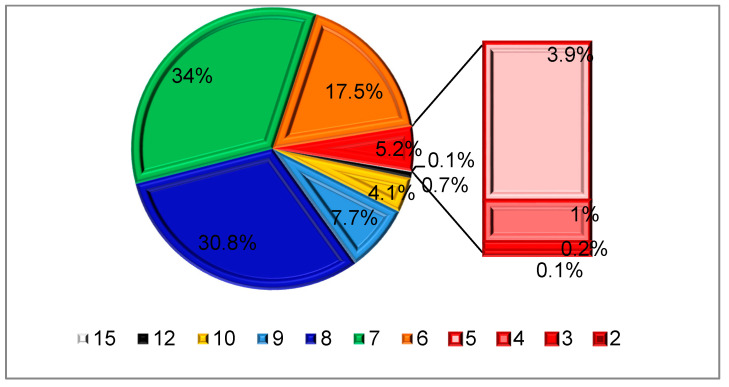
Distribution depending on sleep duration (h).

The distribution of subjects depending on sleep duration is presented on Figure 1. On average, the participants of the epidemiology of cardiovascular diseases in different regions of Russia (ESSE-RF) slept 7.0 (2.0–15.0) hours daily. Overall, 73 respondents (5.2%) reported short sleep <6 h daily, while 69 (4.9%) slept longer than 10 h daily.

The rates of sleep-related complaints are presented in Table 1 and Table 2 (Gender-related differences see in the Supplemental Tables S1 and S2). Short-sleepers significantly more often reported insomnia-related complaints (sleep-onset and/or sleep-maintenance difficulties), the use of hypnotics, and daytime sleepiness. The rates of self-reported snoring and sleep apnea were comparable in subjects with different sleep durations.

A more detailed analysis of the lipid profile (Table 3, gender-related differences see in Supplemental Table S3) demonstrated that short-sleepers had lower levels of Lp(a) and lower rates of increased Lp(a) ≥0.5 g/L, while no differences were found for the levels of other lipids, as well as of ApoAI, ApoB, or their ratio (therefore, they were not included in the further analysis). At the same time, short-sleepers were characterized by the higher values of insulin and insulin resistance assessed by the homeostatic model assessment for insulin resistance (HOMA-IR).

The multinomial logistic regression demonstrated that only the short-sleep duration was independently associated with Lp(a) (significance for the model: χ ^2^ = 41.6, p = 0.003). Other influencing factors were the smoking status and HOMA-IR (Table 4). For long-sleepers, only the female sex appeared to be a predictive factor. When the hypnotics use was added to the multinomial logistic model, the LP(a) level remained an independent factor related to the short-sleep duration, although medication usage is obviously associated with sleep duration (Supplemental Table S4; the results of the analysis with generalized additive model are presented in Supplemental Tables S5 and S6).

## 3. Discussion

Based on the analysis of a large random cohort, we found a significant and independent relationship between short-sleep duration and serum levels of Lp(a). Moreover, in our cohort, short-sleepers showed lower levels of Lp(a) and lower rates of decreased Lp(a) < 5.0 mmol/L.

The available data from epidemiological studies on the association between sleep duration and lipid metabolism disorders is controversial. In the CARDIA study, dyslipidemia (the increase in LDL ≥ 5.0 mmol/L) was registered more often in subjects with shorter sleep durations [12]. On the other hand, the meta-analysis of 153 prospective cohort studies (5,172,710 participants) could not show any association with dyslipidemia due to the lack of data [8]. The same conclusion was made by the research group led by G. Capuccio (2017) [9]. The discrepancy between the published studies and our results can be also explained by the different definitions of short and long-sleep durations applied in various studies. We applied the range from 6 to 9 h daily for a normal sleep duration and considered sleep <6 h as short. In the NHANES study, the lowest risk of metabolic syndrome was found for those sleeping 7 h, although the mean sleep duration in the cohort was below 7 h daily [13]. In addition, in the NHANES study, a U-shape association between the sleep duration and metabolic syndrome was observed. It should be noted that the metabolic syndrome includes both abnormal lipid and glucose metabolisms as the diagnostic criteria. In a different analysis from the NHANES study, the associations of individual lipid parameters and sleep duration were multidirectional [11]. For example, in the analysis of the Japanese Kansai Healthcare Study [10], the sleep duration was categorized as <5 h and from 5 up to 7 h, ≥7 h, which makes difficult the comparisons with other classifications of sleep duration, including our analysis.

Both the NHANES and the China Health and Nutrition Survey demonstrate the gender-related dependence of the association between lipids and sleep [9,13]. According to the data of the China Health and Nutrition Survey (2009) (8574 adults), both shorter and longer sleep were associated with greater risks of abnormal serum lipids only in women [9]. In our cohort, gender did not interact with the association between sleep duration and lipids, although lipids (TC, HDL, and TG) differed significantly in males and females (Supplemental Table S3). However, the majority of the studies did not evaluate Lp(a) [9,10,11,12,13], and in the cohort from the China Health and Nutrition Survey, the Lp(a) levels did not differ among subgroups with different sleep durations [14].

Lp(a) is an atherogenic low-density lipoprotein particle, which is involved in a wide spectrum of physiological processes in normal conditions and in pathology, including angiogenesis, inflammation, endothelial function, etc. Elevated Lp(a) is considered to play an important role in premature cardiovascular pathology [2,15]. The high clinical interest in Lp(a) is also due to the genetic determination of Lp(a) levels, which are not influenced by other commonly assessed confounders, such as diet, anthropometric measures, environmental factors, and concomitant diseases. The target and cut-off values of Lp(a) remain disputable and should be established by future investigations. Although no consensus goal of Lp(a) is established, the general recommendation is to treat other cardiovascular risk factors in patients with high Lp(a) in combination with other hypolipidemic and glucose-lowering drugs [2,15]. At the same time, recent studies have demonstrated that, while increased Lp(a) levels are associated with enhanced atherogenesis, proinflammatory and prothrombotic states [16], a greater risk of cardiovascular events, and poorer prognosis [2,15,17,18], the decreased serum concentrations of Lp(a) are related to the insulin resistance state [19]. These findings suggest that any shift (either to increase or decrease) of Lp(a) might be associated with an unfavorable outcome and should be monitored, so the association between Lp(a) levels and cardiometabolic disease might be U-shaped. Our results indicate that short-sleepers are characterized by both lower Lp(a) and higher insulin concentrations. Moreover, both Lp(a) and insulin resistance assessed by HOMA-IR were significantly associated with short sleep durations but not with long-sleep after an adjustment for various clinical and demographic factors, including self-reported sleep-disordered breathing, which are known to be associated with an increased risk of insulin resistance and its relation to the interaction between Lp(a) and the severity of obstructive sleep apnea (measured by the apnea-hypopnea index) [20].

The large-scale NHANES study demonstrated a U-shaped relationship between sleep duration and the incidence of diabetes mellitus [21]. Even a short-term sleep restriction can cause impaired glucose tolerance [22,23,24]. At the same time, a Taiwanese study reported a different relation, stating that insulin resistance is associated with a longer sleep duration. However, the cohort characteristics (only vegetarians included) and long-sleep definition (>8 h) might explain this discrepancy [25].

A wide spectrum of mechanisms linking glucose metabolism disturbances and short-sleep is being discussed, including sympathetic hyperactivity [26], elevated night and morning cortisol levels associated with decreased peripheral insulin sensitivity [27], increased inflammation, imbalances in appetite-regulating hormones (leptin and ghrelin) [27,28], impaired melatonin secretion, etc. [29]. Moreover, it might be also mediated by common genetic factors, which is confirmed by the studies assessing racial and ethnic differences. Thus, Lp(a) is shown to be significantly higher in Black people, and the racial differences were found to be the most notable for Lp(a) among all the lipid parameters [30].

Interestingly, the significant interpopulation variability and differences in Lp(a) level distribution explained by genetic variants are described. For example, the Finnish population is characterized by lower levels of Lp(a) levels, as well as lower rates of increased Lp(a) above the commonly used thresholds (>30 mg/dl and >50 mg/dl), which correlated with apo(a) isoforms and genetic variants of encoding and regulating genes, including LPA, APOE, and PCSK9 [31]. These data are supported by the GWAS-based meta-analysis [32] and other studies [33] showing the involvement of LPA and APOE isoforms in the regulations of Lp(a) concentration.

Three APOE gene variants—ɛ2, ɛ3, and ɛ4—are investigated with regard to the risks of cardiovascular diseases and metabolic disorders, including dyslipidemia and insulin resistance [34,35,36]. Clinical observations in neurodegenerative diseases evidence the relationship between APOEɛ4 (which is an established genetic factor for Alzheimer’s disease) and sleep disturbances and their progression [36,37]. Thus, G. Tranah et al., based on the results of the Osteoporotic Fractures in Men (MrOS) Study, found that, in subjects with cognitive decline apolipoprotein E (APOE), ε4 genotype carriers (APOE rs429358 SNP) had longer stage 3 NREM-sleep, lower WASO, and higher sleep efficiency (reflecting lower arousability), which the authors explained by the imbalance in neural plasticity and an overactive downscaling of synapses during sleep [37].

We speculate that there are common underlying genetic pathways that link sleep duration and lipid and glucose metabolisms. Sleep patterns and sleep duration are shown (including the genome-wide association study results) to be hereditary, determined and regulated by many genes and their interactions [38]. A number of studies presented contradictory results on the relation between APOE and its variants and sleep disorders [39,40,41]. D.F. Kripke et al. demonstrated an association between the *APOE* variant and sleepiness (assessed by the Epworth Sleepiness Scale), although it had not been prospectively predicted and had low clinical value. However, this study included patients admitted to the sleep lab but not a general population sample and could be biased regarding sleep-related complaints [42].

Surprisingly, very little evidence regarding APOE and sleep duration or other parameters characterizing sleep quality is available. The results of the Baltimore Longitudinal Study of Aging, which included 1264 participants without cognitive impairment, show that the ε4 allele of the APOE gene is associated with the increased probability of having short-sleep (OR = 1.41, 95% CI 1.06–1.88) but not with the sleep-onset or sleep-maintenance problems [43]. Intriguingly, very limited data confirm a relationship between melatonin, which is one of the major regulators of circadian rhythms, circadian genes, and APOE genetic variants [44,45,46], which presents a potential underlying mechanism of the relation between sleep duration and Lp(a) found in our study.

Although our data suggest an independent association between Lp(a) and short-sleep duration, a number of limitations should be considered. The cross-sectional design of the study cannot allow the evaluation of a causal relationship between Lp(a), sleep duration, and insulin resistance. We hypothesized that genetically determined Lp(a) is a predictor associated with sleep duration. However, we cannot rule out the possibility that short-sleep duration is a manifestation of another undiagnosed clinical (neurologic, endocrine, etc.) condition. Another limitation is the subjective assessment of sleep duration based on self-reports. However, self-reported sleep durations significantly correlate with objective (polysomnographic) sleep characteristics [47]. The group of subjects with sleep durations >10 h daily occurred to be too small (10 subjects), so we have included those with self-reported sleep durations ≥10 h daily in the long-sleepers group (so some normal sleepers for whom 10-h sleep durations may be appropriate [45] were considered long-sleepers), which could have also influenced the results. Despite the common use of self-reports for the evaluation of sleep durations, further studies should include the analysis of objectively measured sleep parameters. We should also note a potential limitation related to the measurement of Lp(a) by mass concentration (g/L, as set by our laboratory) instead of molarity. It implies that values could be artificially affected based on the number of Kringle domains within the Apo(a) [48]. In addition, the molar/mass measures of Lp(a) are hardly convertible, which impedes a comparison with the other studies and leads to the confusion in defining reference values [49]. Last, but not least, as no genetic analyses related to lipid and glucose metabolisms were performed within this study, no genetic pathway can be defined. However, our results substantiate the hypothesis about common genetic factors linking sleep duration, the lipid profile, and insulin resistance, and Lp(a) can potentially serve as a surrogate clinical predictor that needs further elucidation in prospective trials and will be assessed in the ongoing follow-up branch of the ESSE-RF study.

## 4. Materials and Methods

The current analysis is based on the data from the St. Petersburg branch of the epidemiological study “Epidemiology of cardiovascular diseases in different regions of Russia (ESSE-RF)”, which was described elsewhere [50,51]. In total, 1600 citizens of St. Petersburg were enrolled and examined within the study. The stratified random sample was formed based on the Kish selection method. All participants signed informed consent and underwent a 12-module survey based on the adapted international questionnaires (the demographics data, lifestyle, medical history, complaints, and therapy) [51]. The survey was completed in the period between 1 November 2012 and 1 May 2013. The protocol was approved by the local ethical committee (approval number #193, 8 October 2012).

The inclusion criterion was ages 25–64 years old. For the present analysis, the exclusion criteria were the following: previously known cardiovascular events, hypolipidemic therapy at the time of the examination, nonavailable/nonvalid responses to the sleep-related questions, nonavailable/nonvalid results of blood tests (lipid profile), and decreased kidney function (estimated glomerular filtration rate < 30 mL/min/1.73 m^2^). Therefore, the data of 1427 subjects were included in the analysis (Table 2 and Table 3).

The module for the assessment of sleep and its disorders included 7 questions.

Sleep duration was assessed by the following question:

(1) How long have you been sleeping daily during the last month?

Numerical answer (hours) was presupposed. We applied the classification of sleep duration proposed by the National Sleep Foundation [52]: normal (recommended) sleep duration—7–9 h, and may be an appropriate normal sleep duration—6–10 h. As in our sample, there were only 11 respondents who reported sleep >10 h daily; we divided the groups as follows: sleep <6 h was considered short, ≥10 h—long, and 6–9 h was assessed as the normal sleep duration.

Four questions evaluated insomnia-related complaints and sleepiness and included several answer options: never; < 1 time per week; 1–2 times per week; and ≥3 times per week.

(2) How often did you have difficulties in falling asleep for ≥30 min after going to bed during the last month?
(3) How often did you have difficulties in falling asleep after midnight awakening during the last month?
(4) How often have you taken sleeping pills during the last month?
(5) How often have you had difficulties in staying awake when required?

Insomnia was considered when sleep-onset (question #2) or sleep-maintenance (question #3) problems were reported occurring ≥3 times per week during the last month [53,54]. The daytime sleepiness (question #5) was considered increased when reported more than 3 times per week [54].

Two questions assessed self-reported sleep-disordered breathing (SDB) with the answer options “Yes”, “No”, and “Do not know”. The diagnosis of SDB was considered probable in the case of an affirmative answer to any of these questions:

(6) “Do you snore?”
(7) “Do you have sleep apnea?”

All subjects underwent a physical examination with the measurements of blood pressure (BP), heart rate (HR), and anthropometric parameters and blood tests.

As for anthropometric measurements, we assessed height (accuracy up to 0.5 cm, in the standing position, by stadiometer Medical RP, TVES, Russia), weight (accuracy up to 100 g, by medical scales HEM-150 MASSA-K, Russia), and waist and hip circumferences (by flexible tape in the standing position, on midway between the lower edge of the rib cage and the iliac crest at the mid-axillary line) and applied the Quetlet equation to calculate body mass index (BMI): weight (kg)/height^2^ (m^2^). Obesity was diagnosed in case of BMI ≥ 30 kg/m^2^.

Among self-reported lifestyle risk factors, we assessed the physical activity level and smoking status. Physical activity was considered low when respondents reported less than 150 min of walking per week (the question “How long (in minutes) do you spend walking per day, including the way to and back from your workplace?”). Smoking status was considered positive in the case of the affirmative answer to the question (“Have you ever smoked (or smoke currently) every day?”).

BP measurements were performed according to the guidelines on the management of arterial hypertension of the European Society of Cardiology/European Society of Hypertension [3]: after 5 min of rest in a supine position, two measurements with 2-min intervals were performed on the right hand (the automatic tonometer “OMRON M3 Expert”, Japan). The mean value was taken for analysis. Subjects were considered hypertensive when systolic BP (SBP) was 140 mmHg and higher and/or diastolic BP (DBP) was 90 mmHg and higher or if they received antihypertensive therapy.

The following fasting blood tests were assessed (laboratory staff was blinded regarding the subjects’ responses): lipid profile, creatinine, glucose, and insulin (Cobas e411 and Cobas Integra 400 plus, Switzerland; reagents from Roche-diagnostics, Germany). The lipid profile included the total cholesterol (TC), high-density lipoproteins (HDL), low-density lipoproteins (LDL), triglycerides (TG), lipoprotein a (Lp(a)), apolipoprotein AI (apoAI), and apolipoprotein B (apoB). Dyslipidemia was considered in cases when the total cholesterol was 6.0 mmol/L and higher and/or low-density lipoprotein was 3.0 mmol/L and higher (none of the subjects included in the analysis took hypolipidemic drugs). Hyperglycemia was diagnosed when the fasting glucose exceeded 6.1 mmol/L; diabetes mellitus was diagnosed either based on previous medical history or if the fasting glucose level was over 7.0 mmol/L [55]. The insulin resistance was assessed by the homeostatic model assessment as a simple tool appropriate for epidemiological studies: HOMA-IR = fasting insulin (microU/L) × fasting glucose (nmol/L)/22.5, with the following correction for the measurement units: HOMA-IR = insulin (pmol/L) × glycemia (mmol/L)/135 [56,57].

The Lp(a) level was considered high when it exceeded 0.5 g/L [2]. The number of subjects with extremely high Lp(a) levels (≥1.8 g/L) composed less than 1%, so this subgroup was not evaluated separately.

The glomerular filtration rate was estimated by the CKD-EPI formula.

For statistical analysis, we used parametric and nonparametric statistics. The Kolmogorov-Smirnov test was applied to assess the distribution of continuous variables. Descriptive statistics included the mean and standard deviation values for the normally distributed variables and median (minimum-maximum) for non-normally distributed variables. For normalization of the non-normally distributed parameters, we applied a log transformation. We applied a frequency analysis (the chi-square) to assess the contingency between the nominal and categorical variables. The continuous variables were compared by one-way analysis of variance (ANOVA). For multiple testing, the Dunnett’s correction was applied. A multinomial logistic regression analysis was used to assess the associations between sleep duration (as the dependent categorical variable: <6, 6–9, and ≥10 h; sleep duration 6–9 h was considered as the reference category) and Lp(a) (as a genetic deterfactor), with adjustments for other potential confounders. Nominal or categorical variables were included as factors (gender, smoking status, physical activity, hypertension, diabetes mellitus, and sleep-disordered breathing), and continuous variables were included as covariates (age, BMI, and HOMA-IR). The results for the estimated potential predictors are presented as the odds ratio (OR) and 95% confidence interval (95% CI). The two-sided *p*-value <0.05 (in the case of multiple testing for 3 groups <0.005) was accepted to define the validity of the statistical hypothesis. The software SPSS 20.0 was used to perform the statistical analysis.

## 5. Conclusions

Based on our results, a short sleep duration is associated with lower Lp(a) levels and higher insulin resistance in the general population, implicating a potential link between sleep duration and adverse cardiometabolic outcomes. These underlying mechanisms and potential common genetic factors require further elucidation in future prospective studies.

## Figures and Tables

**Table 1:** Sleep-related complaints in the cohort. SDB: sleep-disordered breathing.

Sleep Complaint	Whole Cohort, Number (%)	<6 h, Number (% Subgroup)	6–9 h, Number (% Subgroup)	≥ 10 h, Number (% Subgroup)	*p*
Sleep-onset difficulties ≥3 times/week	256 (17.9%)	28 (38.4%)	209 (16.3%)	19 (27.5%)	χ^2^ = 27.4, *p* < 0.001
Sleep-maintenance difficulties (midnight awakenings) ≥3 times/week	176 (12.3%)	21 (28.8%)	142 (11.1%)	12 (17.4%)	χ^2^ = 21.6, *p* < 0.001
Both sleep-onset and sleep-maintenance problems ≥3 times/week	120 (8.4%)	20 (27.4%)	89 (6.9%)	11 (15.8%)	χ^2^ = 42.9, *p* < 0.001
Either sleep-onset or sleep-maintenance problems ≥3 times/week	312 (21.9%)	29 (39.7%)	263 (20.5%)	20 (29.0%)	χ^2^ = 17.2, *p* < 0.001
Daytime sleepiness ≥3 times/week	93 (6.5%)	11 (15.5%)	75 (5.9)	7 (10.1%)	χ^2^ = 11.6, 0.003
Any of insomnia complaints + daytime sleepiness ≥3 times/w	32 (2.2%)	5 (7.0%)	26 (2.0%)	1 (1.4%)	χ^2^ = 7.8, *p* < 0.020
Any of insomnia complaints + short sleep <6h	29 (2%)	29 (39.7%)	0	0	<0.001
Regular snoring	674 (47.2%)	31 (42.5%)	610 (47.5%)	33 (47.8%)	0.70
Sleep apneas	71 (5.0%)	4 (5.5%)	67 (5.2%)	0	0.15
SDB (snoring or sleep apneas)	738 (51.7%)	32 (43.8%)	624 (48.6%)	33 (47.8%)	0.73
Intake of sleeping pills ≥3 times/week	40 (2.8%)	9 (12.3%)	28 (2.2)	3 (4.4%)	χ^2^ = 26.6, *p* < 0.001

**Table 2:** Demographic and clinical characteristics of the groups depending on sleep duration.

Parameter	Whole Cohort M±SD/Number (%)	<6 h M±SD/Number (% Subgroup)	6–9 h M±SD/Number (% Subgroup)	≥ 10 h M±SD/Number (% Subgroup)	*p*
Sex (males), n (%)	501 (35.1%)	28 (38.4%)	459 (35.7%)	14 (20.3%)	χ^2^ = 7.20, *p* = 0.027
Age, years	46.2 ± 11.7	47.1 ± 12.2	46.1 ± 11.7	46.0 ± 11.3	0.67
BMI, kg/m^2^	27.0 ± 5.3	27.6 ± 6.0	27.0 ± 5.2	27.6 ± 6.4	0.52
SBP/DBP, mmHg	129 ± 20/79 ± 12	126 ± 19/78 ± 11	129 ± 20/79 ± 12	127 ± 21/79 ± 13	0.37/0.46
Hypertension*, n (%)	542 (38%)	28 (38.4%)	490 (38.2%)	24 (34.8%)	0.85
Obesity**, n (%)	370 (25.9%)	18 (24.7%)	332 (25.9%)	20 (29.0%)	0.82
Diabetes mellitus***, n (%)	80 (5.6%)	8 (11.0%)	69 (5.4%)	3 (4.3%)	0.12
Dyslipidemia, n (%)	941 (65.9%)	42 (57.5%)	851 (66.2%)	48 (69.6%)	*p* = 0.27
Kidney dysfunction (eGFR < 60 mL/min/1.73 m^2^), n (%)	4 (0.3%)	0	4 (0.3%)	0	0.80
Low physical activity, n (%)	1010 (70.8%)	45 (61.6%)	922 (71.8%)	43 (62.3%)	0.052
Smoking, n (%)	705 (49.4%)	45 (61.6%)	628 (48.9%)	32 (46.4%)	0.09

Footnote: * Hypertension (HTN) considered when systolic blood pressure (BP) (SBP) was 140 mmHg and higher and/or diastolic BP (DBP) was 90 mmHg and higher or if the person received antihypertensive therapy. ** Obesity was diagnosed in the case of BMI≥30 kg/m^2^. *** Diabetes mellitus was considered when known from the previous medical history or if the fasting glucose level was over 7.0. BMI: body mass index and eGFR: estimated glomerular filtration rate.

**Table 3:** Laboratory parameters in the groups depending on sleep duration. HOMA-IR: homeostatic model assessment for insulin resistance, Lp(a): lipoprotein (a), TC: total cholesterol, LDL: low-density lipoproteins, HDL: high-density lipoproteins, TG: triglycerides, ApoAI: apolipoprotein AI, and ApoB: apolipoprotein B.

Parameter	Whole Cohort M±SD/ Me(Min-Max)/ Number (%)	<6 h M±SD/ Me(Min-Max)/ Number (% Subgroup)	6–9 h M±SD/ Me(Min-Max)/ Number (% Subgroup)	≥10 h M±SD/ Me(Min-Max)/ Number (% Subgroup)	*P*
	**Lipid profile**				
TC, mmol/L	5.3 (2.6–10.8)	4.9 (2.6–9.8)	5.3 (2.8–10.8)	5.3 (2.9–8.3)	0.68
LDL, mmol/L	3.3 (0.6–8.0)	3.1 (1.5–6.2)	3.3 (0.6–8.0)	3.4 (1.2–6.1)	0.86
HDL, mmol/L	1.3 (0.5–3.4)	1.2 (0.7–3.4)	1.3 (0.5–2.9)	1.3 (0.8–2.6)	0.22
TG, mmol/L	1.1 (0.2–16.1)	1.2 (0.5–3.8)	1.1 (0.2–16.1)	1.2 (0.5–6.3)	0.39
TG/HDL	1.10 (0.16–17.5)	1.0 (0.26–3.80)	0.81 (0.16–17.5)	0.90 (0.28–7.72)	0.45
Lp(a), g/L	0.10 (0.04–4.50)	0.08 (0.08–1.09)	0.10 (0.04–4.50)	0.15 (0.07–1.75)	0.014 P1-2=0.002 P1-3=0.008 P2-3=0.34
ApoAI, g/L	1.6 (0.3–4.3)	1.44 (0.25–4.19)	1.57 (0,41–4.27)	1.55 (0.32–3.94)	0.071
ApoB, g/L	1.0 (0.2–2.5)	0.98 (0.20–1.77)	1.01 (0.20–2.46)	1.04 (0.20–1.82)	0.77
ApoB/ApoAI	0.65 (0.05–1.65)	0.65 (0.23–1.34)	0.65 (0.05–1.65)	0.66 (0.05–1.40)	0.69
Lp(a)≥0.5, g/L	229 (16%)	4 (5.5%)	207 (16.1%)	18 (26.1)	χ ^2^=11.22, *p* = 0.004
Lp(a) ≥1.8, g/L	10 (0.7%)	0	10 (0.8%)	0	0.57
TC ≥6.0, mmol/L	401 (28.1%)	19 (26%)	360 (28%)	22 (31.9%)	*p* = 0.72
LDL ≥3.0, mmol/L	936 (65.6%)	42 (57.5%)	846 (65.8%)	48 (69.6%)	0.27
	**Glucose metabolism**				
Glucose, mmol/L	5.0 (1.4–14.7)	4.9 (3.6–10.3)	5.1 (1.4–12.9)	5.0 (3.4–14.7)	0.84
Insulin	59.1 (3.0–512.9)	73.4 (6.5–485.3)	58.7 (3.0–512.9)	62.1 (8.2–260.3)	0.001 P1-2 = 0.001 P1-3 = 0.09 P2-3 = 0.81
HOMA-IR	2.22 (0.10–32.2)	2.64 (0.23–26.2)	2.21 (0.10–32.24)	2.31 (0.30–12.67)	0.006 P1-2 = 0.004 P1-3 = 0.19 P2-3 = 0.76
	**Kidney function**				
Creatinine, mcmol/L	67 ± 11	66 ± 10	67 ± 11	65 ± 9	0.21
eGFR, ml/min/1.73 m^2^	103 ± 13	103 ± 13	103 ± 13	103 ± 12	0.87

**Table 4:** The association between sleep duration and other factors (the multinomial logistic regression results). OR: odds ratio, CI: confidence interval, DM: diabetes mellitus.

Group (by Sleep Duration)	Variable	Standard Error	Wald	Degree of Freedom	OR (95%CI)	*p*-value
<6 h	Lp(a)	0.58	4.53	1	0.29 (0.09; 0.91)	0.033
	Sex (male)	0.27	0.006	1	1.02 (0.61; 1.72)	0.94
	Age	0.01	1.42	1	1.02 (0.99; 1.04)	0.23
	BMI	0.03	0.03	1	1.01 (0.95; 1.06)	0.87
	HOMA-IR	0.04	5.17	1	1.09 (1.01; 1.17)	0.023
	Smoking (yes)	0.26	4.66	1	1.76 (1.05; 2.95)	0.031
	Physical activity (low)	0.25	3.18	1	0.64 (0.39; 1.05)	0.07
	HTN (yes)	0.29	0.70	1	0.78 (0.44; 1.39)	0.40
	DM (yes)	0.47	0.47	1	1.38 (0.55; 3.45)	0.49
	SDB (yes)	0.28	2.23	1	0.66 (0.39; 1.14)	0.66
≥10 h	Lp(a)	0.28	2.72	1	1.59 (0.92; 2.77)	0.99
	Sex (male)	0.32	6.34	1	0.44 (0.24; 0.84)	0.012
	Age	0.01	0.53	1	0.99 (0.97; 1.02)	0.47
	BMI	0.03	0.91	1	1.03 (0.97; 1.08)	0.34
	HOMA-IR	0.05	0.76	1	1.04 (0.95; 1.15)	0.38
	Smoking (yes)	0.25	<0.001	1	1.00 (0.60; 1.66)	0.99
	Physical activity (low)	0.26	3.53	1	0.61 (0.37; 1.02)	0.06
	HTN (yes)	0.31	0.45	1	0.82 (0.45; 1.48)	0.50
	DM (yes)	0.68	0.53	1	0.61 (0.16; 2.31)	0.47
	SDB (yes)	0.28	0.01	1	1.03 (0.59; 1.80)	0.92

## Supplementary Materials

Supplementary materials can be found at https://www.mdpi.com/1422-0067/21/13/4680/s1.

## Abbreviations

ApoAI	Apolipoprotein AI
ApoB	Apolipoprotein B
BMI	Body mass index
BP	Blood pressure
DBP	Diastolic blood pressure
DM	Diabetes mellitus
eGFR	Estimated glomerular filtration rate
ESSE-RF	Epidemiology of cardiovascular diseases in different regions of Russia
DL	High-density lipoproteins
HOMA-IR	Homeostatic model assessment for insulin resistance
HTN	Hypertension
LDL	Low-density lipoproteins
Lp(a)	Lipoprotein (a)
SBP	Systolic blood pressure
SDB	Sleep-disordered breathing
TC	Total cholesterol
TG	Triglycerides
