# Supplementary material for: Sleep Duration, Lipid Profile and Insulin Resistance: Potential Role of Lipoprotein(a)
Source: Int J Mol Sci. 2020 Jun 30;21(13):4680. doi: 10.3390/ijms21134680 (PMC7369827; doi:10.3390/ijms21134680)
Supplement: Supplementary file 1 [file ijms-21-04680-s001.pdf]

Table S1. Sleep-related complaints depending on gender [Number (% whole cohort/%row category)]

**Sleep complaint**	**Males (n=501)**	**Females (n=926)**	**P**
Sleep-onset difficulties ≥3 times/week	66 (4.6%/25.8%)	190 (13.3%/74.2%)	**χ^2=11.91, p=0.001**
Sleep-maintenance difficulties (midnight awakenings) ≥3 times/week	36 (2.5%/20.5%)	140 (9.8%/79.5%)	**χ^2=18.92, p<0.001**
Both sleep-onset and sleep-maintenance problems ≥3 times/week	23 (1.6%/19.2%)	97 (6.8%/80.8%)	**χ^2=14.62, p<0.001**
Either sleep-onset or sleep-maintenance problems ≥3 times/week	79 (5.5%/25.3%)	233 (16.3%/74.7%)	**χ^2=16.79, p<0.001**
Daytime sleepiness ≥3 times/week	24 (1.7%/25.8%)	69 (4.9%/74.2%)	χ^2=3.79, p=0.051
Any of insomnia complaints + daytime sleepiness ≥3 times/w	6 (0.4%/18.8%)	26 (1.8%/81.2%)	χ^2=3.85, p=0.050
Any of insomnia complaints + short sleep <6h	10 (0.7%/34.5%)	19 (1.3%/65.5%)	p=0.94
Regular snoring	302 (21.2%/44.8%)	372 (26.1%/55.2%)	**χ^2=52.74, p<0.001**
Sleep apneas	32 (2.2%/45.1%)	39 (2.7%/54.9%)	p=0.07
SDB (snoring or sleep apneas)	197 (13.8%/26.7%)	541 (37.9%/73.3%)	**χ^2=47.51, p<0.001**
Intake of sleeping pills ≥3 times/week	6 (0.4%/15.0%)	34 (2.4%/85.0%)	**χ^2=7.25, p=0.007**
Sleep duration:<br>Short (<6h)<br>Normal (6-9h)<br>Long (≥10h)	 28 (2.0%/38.4%)<br>459 (32.2%/35.7%)<br>14 (1.0%/20.3%)	 45 (3.2%/61.6%)<br>826 (57.9%/64.3%)<br>55 (3.9%/79.7%)	p=0.028

Table S2. Demographic and clinical characteristics of the groups depending on gender.

Parameter	Males (n=501)	Females (n=926)	P
Age, years	44±12	47±11	<0.001
BMI, kg/m²	27.3±4.3	26.9±5.8	0.002
SBP/DBP, mmHg	133±18/82±12	126±21/78±12	<0.001
Hypertension, n (%whole cohort/% raw category)	210 (14.7%/38.7%)	332 (23.3%/61.3%)	**χ^2=5.12, p=0.023**
Obesity, n (%whole cohort/% raw category)	121 (8.5%/32.7%)	249 (17.5%/67.3%)	0.25
Diabetes mellitus, n (%whole cohort/% raw category)	26 (1.8%/32/5%)	54 (3.8%/67.5%)	0.62
Dyslipidemia, n (%whole cohort/% raw category)	344 (24.1%/36.65)	597 (41.8%/63.4%)	0.11
Kidney dysfunction (eGFR<60 ml/min/1.73 m²), n (%whole cohort/% raw category)	2 (0.1%/50%)	2 (0.1%/50%)	0.53
Low physical activity n (%whole cohort/% raw category)	347 (24.3%/34.4%)	663 (46.5%/65.6%)	0.35
Smoking, n (%whole cohort/% raw category)	335 (23.5%/47.5%)	370 (25.9%/52.5%)	**χ^2=94.2, p<0.001**

Table S3. Laboratory parameters in the groups depending on sleep duration**.**

Parameter	Males (n=501)	Females (n=926)	P
		**Lipid profile**	
TC, mmol/l	5.2 (2.6-8.4)	5.3 (2.8-10.8)	0.003
LDL, mmol/l	3.4 (1.2-6.4)	3.3 (0.6-8.0)	0.79
HDL, mmol/l	1.2 (0.6-2.7)	1.4 (0.5-3.4)	<0.001
TG, mmol/l	1.2 (0.4-7.5)	1.0 (0.2-16.1)	<0.001
TG/HDL	1.07 (0.23-8.5)	0.70 (0.16-17.5)	<0.001
Lp(a),g/l	0.09 (0.04-1.70)	0.11 (0.04-4.5)	0.012
ApoAI, g/l	1.4 (0.3-4.3)	1.65 (0.32-4.21)	<0.001
ApoB, g/l	1.0 (0.2-2.0)	1.0 (0.20-2.46)	0.10
ApoB/ApoAI	0.73 (0.05-1.60)	0.61 (0.05-1.65)	<0.001
Lp(a)≥0,5 g/l, n (%whole cohort/% raw category)	75(5.3%/32.8%)	154 (10.8%/67.2%)	0.42
Lp(a) ≥1,8 g/l, n (%whole cohort/% raw category)	1 (0.1%/10%)	9 (0.6%/90%)	0.10
TC ≥6.0 mmol/l, n (%whole cohort/% raw category)	116 (8.1%/28.9%)	285 (20%/71.1%)	χ^2=9.35, p=0.002
LDL ≥3.0 mmol/l, n (%whole cohort/% raw category)	341 (23.9%/36.4%)	595 (41.7%/63.6%)	0.15
		**Glucose metabolism**	
Glucose, mmol/l	5.2 (1.4-12.2)	5.0 (3.1-14.7)	<0.001
Insulin	64.5 (3.0-489.5)	56.4 (6.5-512.9)	0.01

HOMA-IR	2.52 (0.10-18.7)	2.11 (0.22-32.2)	<0.001
		**Kidney function**	
Creatinine, mcmol/l	75±11	62±7	<0.001
eGFR, ml/min/1.73 m^2	107±13	101±12	<0.001

Table S4. The association between sleep duration and other factors, including hypnotics use (the multinomial logistic regression results).

Group (by sleep duration)	Variable	Coefficient	Standard error	z-statistic	OR (95%CI)	p-value
<6 h	**Lp(a)**	-1.25	0.59	-2.13	0.29 (0.09; 0.91)	0.033
	Sex (male)	0.10	0.27	0.36	1.10 (0.65; 1.88)	0.72
	Age	0.01	0.01	0.91	1.01 (0.99; 1.04)	0.36
	BMI	0.01	0.03	0.47	1.01 (0.96; 1.07)	0.64
	**HOMA-IR**	0.10	0.04	2.74	1.10 (1.03; 1.18)	0.0061
	**Smoking (yes)**	0.56	0.26	2.12	1.75 (1.04; 2.95)	0.034
	**Physical activity (low)**	-0.52	0.26	-2.01	0.60 (0.36; 0.99)	0.044
	HTN (yes)	-0.32	0.28	-1.13	0.73 (0.42; 1.26)	0.26
	DM (yes)	0.39	0.60	0.65	1.47 (0.45; 4.78)	0.52
	SDB (yes)	-0.35	0.28	-1.25	0.70 (0.41; 1.22)	0.21
	**Hypnotics**	1.89	0.43	4.39	6.65 (2.85; 15.47)	0.00001
≥10 h	Lp(a)	0.46	0.28	1.65	1.59 (0.92; 2.74)	0.098
	**Sex (male)**	-0.75	0.32	-2.32	0.47 (0.25; 0.89)	0.021
	Age	0.00	0.01	-0.39	1.00 (0.97; 1.02)	0.69
	BMI	0.04	0.03	1.32	1.04 (0.98; 1.09)	0.19
	HOMA-IR	0.04	0.05	0.87	1.04 (0.95; 1.14)	0.38
	Smoking (yes)	-0.01	0.26	-0.03	0.99 (0.59; 1.67)	0.98
	Physical activity (low)	-0.47	0.26	-1.76	0.63 (0.37; 1.05)	0.0778
	**HTN (yes)**	-0.69	0.30	-2.28	0.50 (0.28; 0.91)	0.022
	DM (yes)	0.50	0.77	0.65	1.66 (0.36; 7.51)	0.51
	SDB (yes)	0.11	0.29	0.40	1.12 (0.64; 1.97)	0.69
	Hypnotics	0.83	0.64	1.30	2.29 (0.66; 7.97)	0.19

Table S5. Parameters estimates (GAM) for categorical variables.

Group (by sleep duration)	Variable	Coefficient	Standard error	z-statistic	OR (95%CI)	p-value
<6 h	Sex (male)	0.09	0.27	0.34	1.10 (0.64; 0.64)	0.73
	**Smoking (yes)**	0.58	0.27	2.16	1.78 (1.05; 1.05)	0.031
	Physical activity (low)	-0.49	0.26	-1.91	0.61 (0.37; 0.37)	0.056
	HTN (yes)	-0.33	0.29	-1.14	0.72 (0.41; 0.41)	0.25
	DM (yes)	0.40	0.60	0.66	1.48 (0.46; 0.46)	0.51
	**SDB (yes)**	-0.35	0.28	-1.23	0.71 (0.41; 0.41)	0.22
	**Hypnotics**	1.92	0.44	4.38	6.79 (2.88; 2.88)	0.00001
≥10 h	Sex (male)	-0.73	0.33	-2.24	0.48 (0.25; 0.25)	0.025
	Smoking (yes)	-0.01	0.26	-0.04	0.99 (0.59; 0.59)	0.97
	Physical activity (low)	-0.45	0.26	-1.71	0.64 (0.38; 0.38)	0.087
	**HTN (yes)**	-0.69	0.30	-2.29	0.50 (0.28; 0.28)	0.022
	DM (yes)	0.56	0.77	0.73	1.75 (0.39; 0.39)	0.47
	SDB (yes)	0.13	0.29	0.44	1.13 (0.64; 0.64)	0.66
	Hypnotics	0.82	0.64	1.29	2.27 (0.65; 0.65)	0.19

Presented models were fitted using R version 3.6.3 environment for statistical computing, nnet 7.3-13 (multinomial logistic models) and mgcv 1.8-31 (generalized additive model with multinomial dependent variable) packages. In case of GAM smoothing functions were fitted using restricted maximum likelihood estimator for all quantitative variables.

Table S6. Nonlinear terms (GAM).

Group (by sleep duration)	Variable	Estimated degrees of freedom	χ^2	p-value
<6 h	s[Lp(a)]	1.0001	4.18	0.0408
	s[Age]	3.9329	11.75	0.0313
	s[BMI]	1.0002	0.25	0.62
	s[HOMA-IR]	1.0001	7.24	0.0071
≥10 h	s[Lp(a)]	1.0006	2.86	0.091
	s[Age]	1.0001	0.07	0.79
	s[BMI]	1.5486	2.56	0.33
	s[HOMA-IR]	1.9943	1.45	0.54

Presented models were fitted using R version 3.6.3 environment for statistical computing, nnet 7.3-13 (multinomial logistic models) and mgcv 1.8-31 (generalized additive model with multinomial dependent variable) packages. In case of GAM smoothing functions were fitted using restricted maximum likelihood estimator for all quantitative variables.
